# Majorbio Cloud: An integrated platform for single‐omics and integrated multi‐omics analysis of microbiome and metabolome

**DOI:** 10.1002/imo2.70111

**Published:** 2026-06-19

**Authors:** Jianhua Zhao, Linmeng Liu, Bing Yang, Mengxi Li, Junbiao Zhang, Jichen Han, Xiaodan Li, Yan Wang, Shaobing Du, Qianhui Han, Linna Zhang, Wenjuan Sun, Hao Gao, Yachen Dong, Huasheng Huang, Xianglin Zhang, Kegang Yu

**Affiliations:** ^1^ Shanghai Majorbio Bio‐pharm Technology Co., Ltd Shanghai China

## Abstract

Advances in sequencing and mass spectrometry have propelled life sciences into the multi‐omics era. Metabolomics and microbiomics are widely applied across various research fields, yet massive data analysis faces challenges like limited tool functionality and high technical barriers. The Majorbio Cloud Platform addresses these pain points by establishing four integrated workflows: Untargeted metabolomics, targeted metabolomics, microbiomics, and microbiome‐metabolome integrated analysis, enabling one‐stop analysis from single‐omics to multi‐omics correlation. In a real gut microbiome–metabolome case study, the platform pinpointed core differential microbes and characteristic metabolites, and further annotated functional pathways underlying host phenotypic divergence, demonstrating its reliability for mining biological mechanisms. The platform caters to both novice and experienced researchers with dual‐parameter modes (default/customizable), multi‐user collaboration, standardized pipelines, advanced analytical tools, and one‐click color customization. By simplifying complex analytical procedures and reducing reliance on specialized programming skills, it offers a standardized, reproducible solution for multi‐omics data analysis in related fields.

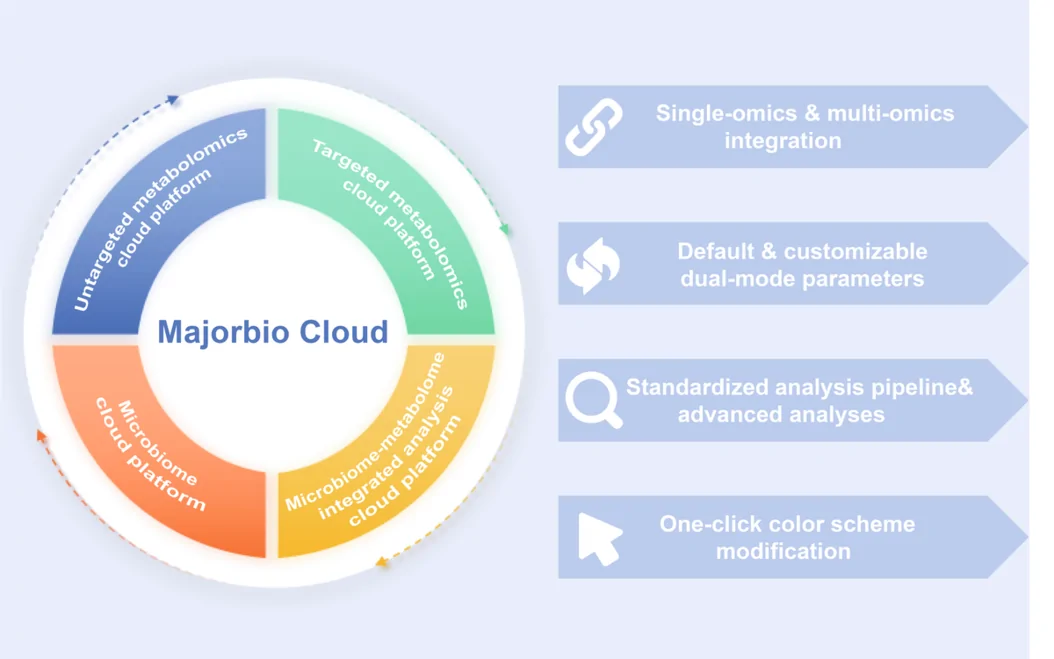

To the editor,

In recent years, driven by the rapid advancement of sequencing technologies, mass spectrometry‐based detection, and artificial intelligence, life science research has transitioned from a single‐omics era to a new epoch characterized by multi‐omics integration. Among these, the combined research of metabolomics and microbiomics has become the core application paradigm of multi‐omics integration. It establishes a link between microbial functional regulation and terminal phenotypic alterations of biological systems, and plays a crucial role in fields such as the regulation of gut microbiota dysbiosis [1], plant–microbe interactions [2], and food fermentation research [3]. Metabolomics, as a key component of systems biology, focuses on the comprehensive identification and quantitative analysis of all small‐molecule metabolites within biological systems. It directly reflects the physiological and pathological states of organisms, holding significant research value and application potential. Metabolomics has demonstrated broad prospects in various fields, including biomarker discovery for diseases [4], food safety control [5], and environmental toxicology [6]. However, both metabolomics research and its integrated analysis with microbiomics are frequently confronted with high data complexity, varied analytical pipelines, and dispersed analytical tools. Although numerous bioinformatics tools and local analytical pipelines currently exist, they generally require substantial computational resources and advanced bioinformatics expertise. Moreover, the lack of effective integration among these tools limits the in‐depth exploration and large‐scale application of both metabolomics and microbiome‐metabolome integrated analysis. In this context, cloud‐based metabolomics analysis platforms have been developed to simplify analytical workflows and improve computational reproducibility via integrated online environments. While some existing platforms (e.g., MetOrigin 2.0 [7], M^2^IA [8], MetaboAnalyst 6.0 [9], MicrobiomeAnalyst 2.0 [10]) have improved the accessibility of metabolomics analysis, most are constructed as independent single‐function systems with fixed analytical pipelines. Even embedded modules such as metabolite source tracking often run as isolated steps instead of being integrated into a full analytical workflow. These platforms are generally limited to narrow analytical scopes and application scenarios—relying merely on standardized pipelines, single‐omics support, or simple microbe–metabolite association analysis—thus lacking a flexible, multifunctional, and customizable framework for personalized multi‐omics analysis (Table S1). To address these limitations, the Majorbio Cloud Platform integrates multiple functions for metabolomics, microbiomics, and cross‐omics analyses. Different from conventional tools with scattered functional modules, our platform embeds metabolite source tracking into a full analytical pipeline covering data preprocessing, functional annotation, traceability interpretation, and multi‐strategy cross‐omics integration, and provides richer algorithm options for microbiome–metabolome association analysis. It not only supports standardized analyses but also allows users to customize parameters based on specific research needs, catering to both novice and experienced researchers. The platform also features multi‐user collaboration and data‐sharing mechanisms, facilitating teamwork and research communication. Collectively, it provides an integrated, interactive analytical solution for metabolomics and related cross‐omics investigations.

Since the launch of Majorbio Cloud Platform in October 2016, it has attracted more than 150,000 scientific research users from over 9000 renowned universities and institutions. To date, the platform has supported the completion of more than 180,000 microbiomics data mining tasks, over 30,000 untargeted metabolomics tasks, more than 3000 targeted metabolomics tasks, and over 8000 microbiome–metabolome correlation analysis tasks. Over the past 5 years, Majorbio Cloud has been explicitly cited in the methodology sections of 11,700 peer‐reviewed journal articles, among which more than 1000 studies involved microbiome–metabolome integrated analysis. These achievements fully demonstrate the practicality and influence of the platform in single‐omics research fields such as microbiomics and metabolomics, as well as multi‐omics integration research, providing robust support for scientific investigations in related disciplines.

## 1 METABOLOMICS AND MICROBIOME–METABOLOME INTEGRATED ANALYSIS WORKFLOW

The Majorbio Cloud Platform is an integrated multi‐omics analysis system built on cloud computing technology, dedicated to providing a standardized online analytical environment for metabolomics, microbiomics, and related cross‐omics research. Since its initial development, the platform has been designed to streamline multi‐omics workflows and reduce the bioinformatics expertise required for data analysis. By integrating functional modules for data processing, statistical analysis, pathway annotation, and machine learning, it enables researchers to focus on biological questions themselves, without being overly burdened by computational implementation details.

The platform supports a wide range of metabolomics workflows, including untargeted, targeted, volatile, and lipidomic metabolomics. This study focuses on the core workflows related to metabolomics and microbiome–metabolome association analysis; detailed descriptions of targeted metabolomics and microbiomics analysis are provided in the Supporting Information. These workflows encompass the complete research pipeline, from broad‐spectrum metabolite screening and accurate quantification of targeted metabolites to cross‐omics mechanistic elucidation, accommodating diverse experimental designs and analytical requirements.

### 1.1 Workflow 1: Untargeted metabolomics cloud platform

Untargeted metabolomics characterizes all small‐molecule metabolites in organisms via separation, identification, and quantification. It explores links between metabolic alterations and physiological, pathological, or phenotypic changes to resolve biological issues. Combined with high‐throughput mass spectrometry profiling, multivariate statistics, and pathway enrichment, it fully reveals metabolic features and functional status of biological systems.

The emergence of the Majorbio untargeted metabolomics cloud platform has significantly advanced the application and development of this research approach. It integrates advanced data analysis algorithms, enabling rapid and accurate processing of massive datasets generated by high‐throughput mass spectrometry. Additionally, it features a user‐friendly interface and streamlined operational workflows, allowing researchers without bioinformatics backgrounds to easily navigate and utilize the platform. The platform only supports qualitative/quantitative data in tabular format. After data upload, all analytical procedures can be completed within a unified framework (Figure 1). The key modules of the latest untargeted metabolomics cloud platform are summarized below (Table S2). (1) Project navigation: Provides step‐by‐step guidance for users to operate the platform. (2) Project analysis summary report: Presents the data processing methods and key results employed in metabolomics research. (3) Application guide: Offers a default comprehensive analytical pipeline that includes all analytical items, while also supporting the construction of customized simplified pipelines to exclude irrelevant analyses, resulting in a cleaner and more focused analytical interface. (4) Project background: Displays relevant contextual information associated with the research project. (5) Data preprocessing: The raw data were processed through a series of procedures, including missing value filtering (by default, feature peaks with a missing value ratio greater than 20% in each group were removed), missing value imputation (by default, imputed with the minimum value of all samples), data normalization (total sum normalization by default), quality control validation (only variables with RSD < 30% were retained by default), and log transformation (Log_10_ transformation by default). This process reduces the impact of irrelevant variables in the dataset, facilitating the screening and analysis of potential target differential metabolites. (6) Sample comparative analysis: Evaluates the reproducibility of intra‐group samples and verifies whether inter‐group differences meet expected outcomes. (7) Metabolite annotation information: In addition to annotating identified metabolites based on the Kyoto Encyclopedia of Genes and Genomes (KEGG) and Human Metabolome Database (HMDB) databases, this version newly adds metabolite source tracing analysis (Figure 1). Relying on self‐established animal, plant, and microbial metabolite databases (Table S3), this analysis can accurately trace and classify metabolite origins in fecal and other host–microbe interaction samples into three categories (host‐derived, microbial‐derived, and host–microbe co‐metabolized), providing an important biological basis for subsequent microbiome data interpretation and integrated analysis of the two omics. (8) Analysis of differential metabolites: Differential metabolites between groups were screened by integrating variable importance in projection (VIP) values (by default, VIP > 1) derived from multivariate statistical analysis with fold change (by default, FC > 1 or FC < 1) and *p*‐value (by default, *p* < 0.05) obtained from univariate statistical analysis. (9) Metabolite set analysis: Conducts KEGG pathway annotation and enrichment analysis on the screened metabolite sets to identify key regulatory pathways. (10) Batch color scheme modification: Offers 33 mainstream journal‐compliant color schemes, enabling one‐click configuration of color schemes and image fonts. Additionally, this platform provides over 10 advanced analytical methods, including Mendelian randomization, for in‐depth data exploration. Based on the standard analysis pipeline, we have also introduced “Extensions” [11]. Users can directly employ the data tables generated by the pipeline and perform customized data mining via flexible parameter configuration, which substantially improves data utilization efficiency and analytical flexibility.

**Figure 1:**
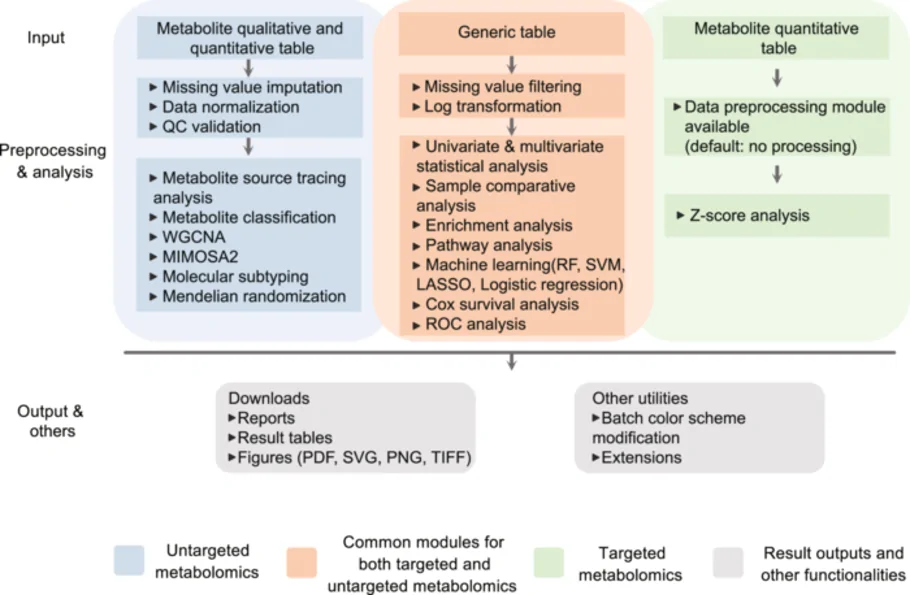
Untargeted metabolomics and targeted metabolomics cloud platform analysis workflow. This platform supports the upload of metabolite qualitative and quantitative tables. The orange modules represent data preprocessing and analysis workflows shared by both untargeted and targeted metabolomics. The blue modules correspond to analytical procedures specific to untargeted metabolomics, including advanced analyses such as metabolite source tracking, weighted gene co‐expression network analysis (WGCNA), and Mendelian randomization. The green modules are dedicated to analytical workflows unique to targeted metabolomics. All pipelines generate downloadable reports, result tables, and publication‐ready visualizations, with additional utilities such as batch color scheme modification and extensions available.

The Majorbio untargeted metabolomics cloud platform, through its systematic and automated analytical workflows, builds a bridge from data to biological discoveries. Leveraging standardized analytical modules and advanced data mining tools, it provides researchers with a comprehensive solution encompassing data quality control, differential metabolite identification, and mechanistic interpretation. This platform not only significantly lowers the technical barriers and time costs associated with metabolomics research but also effectively enhances the depth of data interpretation and the efficiency of scientific output.

### 1.2 Workflow 2: Microbiome–metabolome integrated analysis cloud platform

The integration of microbiomics and metabolomics enables the investigation of phenotypic changes potentially induced by alterations in the host microbial community structure, as well as the understanding of how microbial communities influence the metabolic status of the environment or the host through microbial metabolism and co‐metabolism with the host. Currently, this integrated approach has been widely applied in various research fields.

The microbiome–metabolome integrated analysis cloud platform is equipped with dual workflows, supporting multiple omics data analysis combinations, and enabling integrated analysis of microbial diversity/metagenomics with untargeted metabolomics/targeted metabolomics. Through multi‐omics analysis, it integrates microbiomics and metabolomics data to comprehensively elaborate on and solve biological problems at the levels of species, genes, metabolites, and metabolic pathways. The microbiome–metabolome integrated analysis cloud platform mainly includes the following analytical modules (Figure S1): (1) Overall consistency analysis: It is used to analyze the similarities and variations of samples between microbiome and metabolome, and to evaluate the consistency of microbiome and metabolome results at the overall level. (2) Correlation analysis: This analysis is performed on interested or differential species and metabolites, including seven analytical items such as Mantel test network heatmap, correlation heatmap, chord diagram, and canonical correspondence analysis (CCA). (3) Biomarker panel screening: This module uses machine learning methods (e.g., random forest, logistic regression, etc.) to screen key biomarkers from microbial, metabolite, and association factor data (Table S2). (4) Pathway annotation analysis: When conducting association analysis between metagenomics and metabolomics, this module can generate customized functional pathway maps. It integrates metagenomics and metabolomics data based on KEGG pathways to identify species and metabolites with significant changes involved in the same biological process, enabling rapid identification of key species. Meanwhile, the KEGG functional potential of the species can be obtained.

In summary, the Majorbio microbiome–metabolome integrated analysis cloud platform achieves in‐depth correlation and systematic integration of microbiome and metabolome data through a multi‐workflow integrated analysis framework. It reveals the statistical associations among “species‐function‐metabolites,” promoting research from correlation description to the exploration of causal mechanisms and biomarkers.

## 2 CASE STUDY

To showcase the analytical capability of Majorbio Cloud Platform for metabolomics and microbiomics data, we leveraged a recent study on the gut microbiome and fecal metabolomics of Gansu red deer (*Cervus elaphus kansuensis*) [12]. This study aimed to compare differences in gut microbiota structure, diversity, and metabolic profiles between Gansu wild red deer (WRD) and captive red deer (CRD). Both 16S rRNA marker gene sequencing and LC‐MS/MS‐based metabolomics were performed. Using the accession numbers provided in the original study, we downloaded raw sequencing data from the NCBI Sequence Read Archive database and metabolite concentration tables from MetaboLights. Raw data processing was performed via our DADA2 pipeline to obtain the ASV abundance table and taxonomic annotations. The ASV abundance table and metabolite expression matrix were separately uploaded to the Majorbio Cloud Platform for single‐omics analysis and integrative analysis.

In metabolomics analysis, based on metabolite source tracing analysis, a total of 989 metabolites were classified into four groups: 314 host (animal) metabolites, 332 microbial metabolites and 227 co‐metabolism metabolites, and 570 metabolites of unknown origin (Figure 2A). The HMDB classification annotation results revealed that “lipids and functional‐like molecules” annotated the most metabolites, followed by “organoheterocyclic compounds,” “benzenoids,” and “organic acids and derivatives” (Figure 2B). Plots of orthogonal partial least squares discriminant analysis (OPLS‐DA) scores revealed significant differences between the CRD and WRD groups, indicating that fecal metabolites in Gansu red deer were altered in a remarkable manner (Figure 2C). 402 differential metabolites (VIP > 1 and *p* < 0.05) were detected in the CRD versus WRD, of which 154 metabolites were decreased, and 248 metabolites were increased. Specifically, compared with the WRD group, the levels of anacardic acid, 2‐[2‐(2‐pyridyloxy)ethoxy]pyridine, O‐Desmethylnaproxen, harmine, and FAHFA (2:0/23:0) were significantly increased in CRD (*p* < 0.05), while the levels of MGMG (18:2), prostaglandin A3, and protectin D1 were significantly decreased in the CRD group (*p* < 0.05) (Figure 2D). KEGG pathway enrichment analysis further showed that the differential metabolites were mainly significantly enriched in biosynthesis of unsaturated fatty acids, tyrosine metabolism, phenylalanine metabolism, linoleic acid metabolism, TCA cycle, and valine, leucine, and isoleucine degradation (Figure 2E). The results of 16S rRNA gene sequencing are shown in the Supplementary Materials.

**Figure 2:**
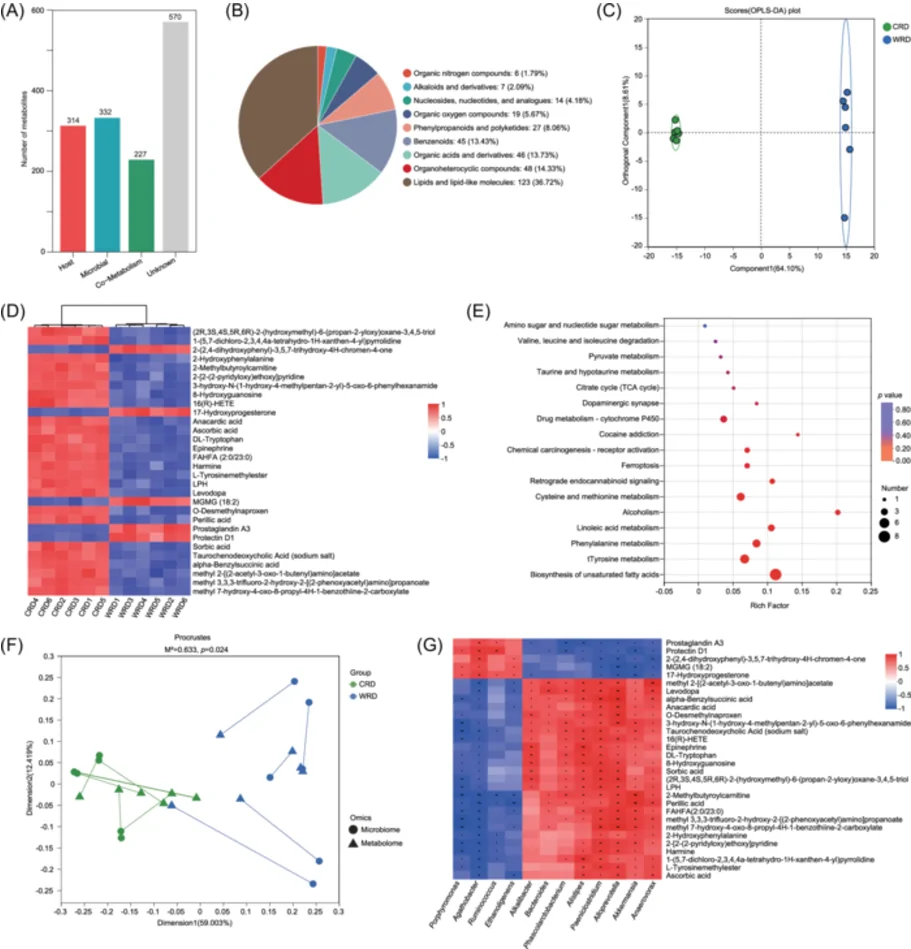
Fecal metabolomics and integrative analysis results of wild red deer (WRD) and captive red deer (CRD) of *Cervus elaphus kansuensis*. (A) Source tracing analysis results of all metabolites. (B) HMDB annotation of all metabolites. (C) Orthogonal partial least squares discriminant analysis (OPLS‐DA) score scatter plot. (D) Expression clustering heatmap of differential metabolites. (E) KEGG pathway enrichment bubble plot. (F) Procrustes analysis. (G) Spearman correlation analysis between differential bacterial genera and differential fecal metabolites. Significance levels were defined as follows: * for *p* < 0.05, ** for *p* < 0.01, and *** for *p* < 0.001.

Procrustes analysis and Spearman correlation analysis were performed to further explore the relationship between gut microbiota and fecal metabolites. As shown in Figure 2F, procrustes analysis revealed a strong overall association between gut microbiota and the fecal metabolome (*p* < 0.05). Spearman correlation analysis was performed between differential bacterial genera and differential fecal metabolites (Figure 2G). Differential metabolites were ranked by *p*‐value in ascending order, and the top 30 metabolites were selected for correlation analysis. The results indicated that most differential bacterial genera exhibited significant correlations with differential metabolites. For example, *Alloprevotella* showed a significant positive correlation with 2‐[2‐(2‐pyridyloxy)ethoxy]pyridine, harmine, LPH, FAHFA (2:0/23:0), sorbic acid, and ascorbic acid. The above analysis results showed a high consistency with those of the original study.

Several previous studies [11, 13, 14] have introduced basic microbiome and metabolome functions of Majorbio Cloud. This work highlights its novel contributions: we systematically describe the metabolite source tracking module for discriminating microbe‐derived and host‐derived metabolites, provide complete methodological details, and conduct qualitative benchmarking against mainstream platforms. Real multi‐omics data validation further confirms its reliability and reproducibility.

However, the current version of the platform has certain limitations: the machine‐learning module supports only mainstream algorithms, and although the platform covers most common analytical tasks, highly customized multi‐omics analyses may still require additional script‐based processing. These limitations will be addressed in future platform updates.

In summary, Majorbio Cloud is a unified, interactive, and scalable one‐stop platform for microbiome, metabolome, and multi‐omics integration analysis. It lowers bioinformatics barriers for novice users and supports flexible customization for advanced researchers. This platform helps accelerate multi‐omics research and deepen mechanistic insights into complex biological systems.

## AUTHOR CONTRIBUTIONS

**Jianhua Zhao**: Conceptualization; investigation; methodology; supervision; writing—original draft; writing—review and editing. **Linmeng Liu**: Conceptualization; investigation; methodology; software; data curation; validation; formal analysis; visualization. **Bing Yang**: Conceptualization, data curation; investigation; validation; formal analysis. **Mengxi Li**: Methodology; data curation; investigation; validation; formal analysis; visualization; writing—original draft. **Junbiao Zhang**: Methodology; data curation; investigation; validation; formal analysis; visualization; writing—review and editing. **Jichen Han**: Conceptualization; methodology; data curation; investigation; validation; formal analysis; supervision; visualization; project administration; resources. **Xiaodan Li**: Methodology; data curation; investigation; validation; formal analysis; visualization. **Yan Wang**: Methodology; data curation; investigation; validation; formal analysis; visualization. **Shaobing Du**: Methodology; data curation; investigation; validation; formal analysis; visualization. **Qianhui Han**: Methodology; data curation; investigation; validation; formal analysis; visualization. **Linna Zhang**: Methodology; data curation; investigation; validation; formal analysis; visualization. **Wenjuan Sun**: Methodology; data curation; investigation; validation; formal analysis; visualization. **Hao Gao:** Software; data curation; investigation; validation; formal analysis. **Yachen Dong**: Methodology; data curation; investigation; validation; formal analysis; visualization. **Huasheng Huang**: Resources. **Xianglin Zhang**: Resources. **Kegang Yu**: Resources. All authors contributed to the development of Majorbio Cloud.

## CONFLICT OF INTEREST STATEMENT

The platform described in this manuscript is related to an authorized patent ZL201810796979.6. The authors are employees of Majorbio. There are no other competing interests to declare.

## DATA SECURITY AND PRIVACY POLICY

All raw data and intermediate analysis files are stored securely in our cloud infrastructure, with strict access controls and encryption protocols in place. Users retain full ownership and ultimate management authority over their data. Through the platform's interface, they can manage datasets, control sharing, and assign granular permissions to collaborators. For academic and institutional researchers, the platform provides standardized paid services with clear terms of service, and offers differentiated analytical standards corresponding to different payment tiers.

## AI STATEMENT

Doubao (ByteDance) was used for English language polishing. All manuscript content has been fully reviewed and verified for accuracy by the authors.

## ETHICS STATEMENT

No animals or humans were involved in this study.

## Supporting information


**Table S1:** Systematic comparison of Majorbio Cloud with other platforms.
**Table S2:** Software and packages for metabolomics and microbiome–metabolome association analysis.
**Table S3:** Information on databases related to metabolite source tracking analysis.

**Figure S1:** Microbiome‐metabolome integrated analysis workflow.
**Figure S2:** 16S rRNA gene sequencing analysis results of gut microbiome in Gansu red deer.

## Data Availability

All data are available at https://cloud.majorbio.com/. Supplementary materials (results, figures, tables, graphical abstract, slides, videos, Chinese translated version, and updated materials) may be found in the online DOI or iMeta Science http://www.imeta.science/imetaomics/.
