# Supplementary material for: Majorbio Cloud: An integrated platform for single‐omics and integrated multi‐omics analysis of microbiome and metabolome
Source: IMetaOmics. 2026 Jun 19;3(3):e70111. doi: 10.1002/imo2.70111 (PMC13674410; doi:10.1002/imo2.70111)
Supplement: Supplementary file 1 [file IMO2-3-e70111-s001.docx]

**Supporting information to**

**Majorbio Cloud: An integrated platform for single-omics and integrated multi-omics analysis of microbiome and metabolome**

**Running Title: Majorbio Cloud: A platform for metabolome and microbiome analysis**

Jianhua Zhao^#^*, Linmeng Liu^#^, Bing Yang^#^, Mengxi Li^#^, Junbiao Zhang^#^, Jichen Han^#^*, Xiaodan Li, Yan Wang, Shaobing Du, Qianhui Han, Linna Zhang, Wenjuan Sun, Hao Gao, Yachen Dong, Huasheng Huang, Xianglin Zhang, Kegang Yu

Shanghai Majorbio Bio-pharm Technology Co., Ltd, Shanghai 200120, China

^#^These authors contributed equally: Jianhua Zhao, Linmeng Liu, Bing Yang, Mengxi Li, Junbiao Zhang and Jichen Han

^*^Correspondence: [jichen.han@majorbio.com](mailto:jichen.han@majorbio.com) (Jichen Han), [jianhua.zhao@majorbio.com](mailto:jianhua.zhao@majorbio.com) (Jianhua Zhao)

**Workflow 3: Targeted metabolomics cloud platform**

As an important branch of metabolomics, targeted metabolomics plays a crucial role in fields such as life sciences, medical research, and precision medicine, boasting broad application prospects. Targeted metabolomics is a research and analytical technique focusing on specific metabolites. Typically, it employs ultra-performance liquid chromatography (UPLC) or gas chromatography (GC) combined with high-sensitivity mass spectrometers for the specific detection and analysis of target compounds, and calculates the absolute content of substances based on standard curves constructed from standard samples of different concentrations. The number of metabolites detected by targeted metabolomics is usually relatively limited, leaving many researchers at a loss regarding subsequent data mining. The Majorbio targeted metabolomics cloud platform enables one-click data preprocessing, which can reduce the impact of variations irrelevant to the research objectives in the data and obtain data conducive to analysis (Figure **1**). Subsequently, sample comparative analysis is performed to intuitively display the overall status of samples and the expression trend of metabolites in the samples. Then, pathway annotation analysis is conducted for all metabolites to identify those involved in the same biological process, and the KEGG functional potential of metabolites can be obtained simultaneously. The candidate biomarker screening module can be used to screen differential metabolites between groups. For the screened key metabolites, metabolite correlation analysis can be performed to further understand the mutual regulatory relationships between metabolites during the process of biological state changes. Metabolite correlation often reveals the synergy of changes between metabolites. In addition, Z-score analysis and VIP (Variable Importance in Projection) value analysis can be used to intuitively display the expression trend changes and importance of differential metabolites. If the number of samples exceeds 30, advanced analyses such as machine learning can also be performed for in-depth data mining (Table **S2**).

Therefore, the core value of the Majorbio targeted metabolomics cloud platform lies in that it systematically addresses the common challenge of researchers being "at a loss where to start" when faced with targeted data through an intelligent analytical workflow. This workflow combines the advantages of high throughput and high sensitivity of targeted metabolomics with in-depth data mining capabilities, making it a key bridge connecting precise quantitative data and complex biological mechanisms.

While metabolomics captures phenotypic metabolic changes, microbiome composition and function are often the key drivers of observed metabolic shifts. To support fully integrated multi-omics research, we established a comprehensive microbiome analysis workflow as a foundation for cross-omics integration.

**Workflow 4: Microbiome cloud platform**

The microbiome cloud platform provides a comprehensive set of analytical workflows, covering microbial diversity, metagenomics, metatranscriptomics, microbial genome analysis, among others (as detailed in [**1**]). This platform enables one-stop, multi-dimensional microbiome data analysis, laying a solid foundation for subsequent in-depth multi-omics association studies.

With robust single-omics workflows in place for both metabolome and microbiome, the final and most critical step is to systematically link microbial community structure, function, and metabolic phenotypes. We therefore designed a dedicated microbiome-metabolome integrated analysis workflow to reveal core biological mechanisms.

**Detailed descriptions of the results of 16S rRNA gene sequencing of Gansu red deer gut microbiome in the case study**

For 16S rRNA sequencing, alpha diversity analysis showed that the Good’s coverage index of CRD and WRD was 0.99969 and 0.99978, respectively. This indicated that an average of 99.90% of microbial taxa were annotated, confirming that the sequencing data sufficiently captured the bacterial composition of the samples and adequately reflected their actual microbial community characteristics. The Chao1 index and Shannon diversity index were applied to evaluate species richness and community diversity, respectively. The CRD group exhibited a higher Chao1 and Shannon value than the WRD group, suggesting a greater number of low-abundance species and higher community diversity in CRD (Figure **S2A,B**). Then, *Firmicutes*, *Bacteroidetes*, and *Pseudomonadota* were the dominant bacterial phyla in both CRD and WRD (Figure **S2C**). At the genus level, *Intestinimonas*, *Phocaeicola*, *Acetivibrio*, and *Pseudoflavonifractor* were dominant genera in both CRD and WRD (Figure **S2D**). Differential analysis was performed on the top 50 genera in abundance, as shown in Figure **S1E**, compared with the WRD group, *Alistipes*, *Bacteroides*, *Akkermansia*, *Phascolarctobacterium*, *Paeniclostridium* and *Anaerovorax* had significantly increased abundance in the CRD group compared with the WRD group, whereas, *Porphyromonas*, *Ruminococcus*, *Ethanoligenens*, and *Agathobacter* had significantly decreased abundance in the CRD group compared with the WRD group.

**FIGURE S1 Microbiome-metabolome integrated analysis workflow.** The first three parts are general integration analysis workflows for microbial diversity/metagenomics with untargeted metabolomics/targeted metabolomics, and the fourth part is the specific integration analysis workflow unique to metagenomics with untargeted metabolomics/targeted metabolomics.

**FIGURE S2 16S rRNA gene sequencing analysis results of gut microbiome in Gansu red deer.** (A) Chao1 index. (B) Shannon index. (C) Bar plot of gut microbiota at phylum level. (D) Bar plot of gut microbiota at genus level. (E) Differential of gut microbiota at genus level.

**REFERENCES**

1. Zhao, Jianhua, Linmeng Liu, Jichen Han, Junbiao Zhang, Xiaodan Li, Yan Wang, Qianhui Han, et al. 2026. “Majorbio Cloud 2026 provides comprehensive analysis workflows for microbiome.” *iMeta* 5: e70109. https://doi.org/10.1002/imt2.70109
